# Revealing the Effect of Ni Alloying on the Ion Irradiation Response of Cr Coatings

**DOI:** 10.3390/ma19153262

**Published:** 2026-08-01

**Authors:** Changfeng Dong, An Li, Hongyang Xin, Tao Peng, Zhien Ning, Dongsheng Xie, Jiaxuan Si, Wei Zhang, Changqing Teng, Xiaoyong Wu

**Affiliations:** 1 The First Sub-Institute, Nuclear Power Institute of China, Chengdu 610041, China; xinhy@npic.ac.cn (H.X.); pengtaoustb@163.com (T.P.); np210006@163.com (Z.N.); xds24240808@163.com (D.X.); sjx@npic.ac.cn (J.S.); zw812@npic.ac.cn (W.Z.); tcg@npic.ac.cn (C.T.); 2 The Fourth Sub-Institute, Nuclear Power Institute of China, Chengdu 610041, China; la1783728496@163.com

**Keywords:** CrNix coating, ion irradiation, microstructure evolution, mechanical properties

## Abstract

**Highlights:**

- CrNi coatings with 6 MeV Au ion irradiation with varied Ni contents show changed morphology and microstructure: Low-Ni coatings keep good crystallinity, while 27% Ni coating presents severe structural disorder and degraded crystallinity.
- Post-irradiation microstructures differ greatly: Low-Ni coatings form massive dislocation loops with weakened diffraction peaks; the 27% Ni coating has scarce dislocation loops and strengthened diffraction signals. Its initial disordered structure undergoes irradiation-induced crystallization via ion thermal spike and collision cascade effects.
- Unirradiated coating hardness peaks at 12.7 GPa (17% Ni) from grain refinement and solid-solution strengthening, and drops at 27% Ni due to poor crystallinity. After irradiation, hardness reaches 13.4 GPa at 17% Ni and declines to 10.7 GPa with irradiation softening at 27% Ni; 17% Ni alloying yields optimal irradiation hardening.

**Abstract:**

Chromium coatings with excellent corrosion resistance and strong Zr interfacial bonding are economical candidates for accident-tolerant fuel claddings. Irradiation-triggered elemental interdiffusion and interfacial voids severely degrade their service reliability. Ni alloying was introduced into the Cr matrix to obtain composite coatings with improved mechanical properties and irradiation resistance. CrNi coatings with different Ni contents were deposited using magnetron sputtering, whose microstructural features, phase composition, mechanical properties and irradiation behavior were comprehensively characterized by XRD, SEM, TEM and mechanical measurements. The pristine CrNi coatings display compact and uniform microstructural morphologies. Increasing Ni concentration significantly refines the columnar grain architecture and diminishes grain dimensions. Post-irradiation microstructural characterization reveals distinct structural evolution features of CrNi coatings with different Ni contents. Pure Cr and low-Ni coatings present enhanced XRD diffraction intensities and contain high-density irradiation-induced dislocation loops. The 27 at.% Ni coating after irradiation is indicative of irradiation-triggered local recrystallization and defect annihilation. Mechanical tests confirm that moderate Ni alloying (~17 at.%) achieves improved resistance to irradiation-induced hardening through solute–defect interaction effects, whereas excessive Ni (~27 at.%) degrades mechanical properties owing to aggravated lattice disorder, increased free volume, and soft Ni-phase dilution effects.

## 1. Introduction

Zirconium alloys have excellent corrosion resistance to high-temperature and high-pressure water, favorable mechanical properties and a low thermal neutron absorption cross-section, and have been successfully employed as fuel-cladding materials in light water reactors (LWRs) for over 50 years [1,2]. Nevertheless, the Fukushima nuclear accident in 2011 prompted the global nuclear power community to reassess the safety performance of zirconium cladding. Accordingly, accident-tolerant fuels (ATFs) have been proposed and extensively investigated [3,4,5,6,7]. The candidate cladding materials for ATF mainly include FeCrAl [8], molybdenum alloys [9], SiC [10], MAX phases [11] and various coatings [12,13]. The research and verification of novel alternative claddings require a long cycle. By contrast, coated zirconium alloys can retain the inherent advantages of the substrate, which makes them easier to realize engineering applications in the short and medium term with promising development prospects.

Particularly, Cr coatings possess excellent high-temperature oxidation resistance and corrosion resistance, and are considered the most engineering-feasible solution at present. Muktanova et al. [14] studied the protective effect of multilayer Cr coatings on E110 zirconium alloy under 1100 °C steam oxidation, confirming that it forms a dense Cr_2_O_3_ barrier to inhibit oxidation of the Zr substrate. Wei et al. [15] showed that after 3000 h corrosion in simulated reactor coolant, Cr/Zr-4 samples had 1/7 the mass gain of uncoated ones, with significantly improved corrosion resistance. Chen et al. [16] investigated thick Cr coatings on Zr-4 alloy under thermal shock at 1000–1300 °C, confirming the coatings maintain superior thermal shock resistance and interfacial adhesion even at 1300 °C. In addition, several studies have reported favorable irradiation resistance of Cr coatings under specific ion-irradiation conditions. Dabney et al. [17] focused on the microstructure evolution of cold-sprayed Cr coatings on zirconium alloy under 20 MeV Kr^8+^ ion irradiation, confirming that the Cr/Zr interface maintains microstructural stability at approximately 10 dpa. Wang et al. [18] conducted low-dose Xe^20+^ irradiation on magnetron-sputtered Cr coatings, and the intact surface indicated excellent irradiation tolerance.

Despite the above-mentioned advantages demonstrated in short-term or low-dose simulated environments, recent studies indicate that pure Cr coatings still face several challenges under long-term normal operation and potential irradiation conditions in light water reactors (LWRs), particularly when considering synergistic effects. First, the coefficient of thermal expansion (CTE) mismatch between Cr and the Zr alloy can induce significant residual stress at the interface, which may lead to coating delamination [19,20,21]. Second, neutron irradiation can cause irradiation-induced hardening and embrittlement in pure Cr coatings, primarily due to the formation and accumulation of point defects, dislocation loops, and void-like defects. Third, under irradiation-enhanced diffusion and in the absence of a continuous, protective oxide layer, the interaction between Cr and Zr may promote interdiffusion and the formation of brittle intermetallic phases (e.g., Cr2Zr), thereby degrading the overall integrity of the cladding system.

In response to these limitations, alloying strategies have been proposed to tailor the microstructure and mechanical properties of Cr-based coatings. Nickel, as a common alloying element in nuclear materials, exhibits several beneficial characteristics when incorporated into Cr. Ni has a face-centered cubic structure and can form solid solutions with Cr over a wide composition range. Moreover, Ni additions have been shown to refine grain size, reduce residual stresses, and improve the ductility of Cr-based alloys, all of which are desirable for mitigating irradiation-induced embrittlement. Accordingly, Ni-Cr coatings have long been extensively adopted as bond coats for thermal barrier coatings [22]. Julian et al. demonstrated that CrNi alloys possess high hardness and favorable wear resistance [23]. Dmitrii et al. deposited NiCr and Cr coatings on Zr-1Nb alloy substrates via magnetron sputtering [24]. The results revealed that as-deposited Ni-Cr coatings exhibit superior mechanical properties compared with pure Cr coatings. NiCr alloy coatings also serve as high-temperature oxidation-resistant layers for material protection, and those with low Ni content present better high-temperature oxidation performance. Additionally, CrNi alloys are widely utilized in anti-corrosion modification of metallic materials owing to their excellent corrosion resistance. Zhang et al. fabricated Cr-Ni-N coatings by magnetron sputtering and revealed that Ni addition significantly promotes the preferred orientation growth of crystal planes [25]. To date, systematic research on irradiation effects of CrNi coatings is relatively scarce, but Ni is known to enhance defect recombination by introducing local lattice distortion and modifying the migration behavior of point defects [26,27], making relevant irradiation investigations highly valuable and indispensable.

On this basis, systematic explorations are carried out on Au-ion irradiation behavior of CrNi alloy coatings with variable Ni addition. Microstructural evolution and mechanical performance alteration upon irradiation are comprehensively examined through nanoindentation measurements. This work aims to provide a fundamental understanding of the role of Ni in enhancing the performance of Cr-based ATF coatings and to offer practical guidance for the design of advanced coatings for next-generation LWRs. Three core research objectives are defined as follows:

(1) To reveal the influence of Ni content on the as-deposited microstructure, grain morphology and intrinsic mechanical properties of magnetron-sputtered CrNi coatings.
(2) To clarify the distinct microstructural evolution mechanisms of CrNi coatings with different Ni concentrations under 6 MeV Au ion irradiation, including the irradiation-induced crystallization phenomenon observed in high-Ni coatings.
(3) To quantify the irradiation hardening/softening behavior of CrNi coatings and identify the optimal Ni addition for superior resistance to irradiation-induced hardening.

## 2. Experimental Section

### 2.1. Sample Preparation

CrNi coatings with varying Ni contents were deposited onto Zr-4 alloy substrates using radio-frequency (RF) magnetron sputtering. The Zr substrates were cut into 10 mm × 10 mm squares with a thickness of 1.5 mm, followed by mechanical grinding and polishing. The specimens were first ground using silicon carbide abrasive papers of progressively finer grits up to P3000, and then mechanically polished with diamond suspension to yield a scratch-free mirror surface. Prior to deposition, the substrates were ultrasonically cleaned in acetone and ethanol, respectively. A composite sputtering target was constructed by assembling high-purity Cr (≥99.99%) and high-purity Ni chips (≥99.99%). The Ni content in the coatings was controlled by varying the number of Ni chips attached to the Cr target. During deposition, the base pressure of the sputtering chamber was maintained below 5 × 10^−3^ Pa, and the substrate temperature was held at approximately 300 °C. High-purity argon was introduced at a constant flow rate of 55 sccm, resulting in a sputtering pressure of about 0.52 Pa. The sputtering power applied to the composite target was fixed at 200 W. By adjusting the relative area ratio of Cr to Ni chips, four distinct CrNi coatings with Ni atomic percentages of 0%, 9%, 17%, and 27% were synthesized. For simplicity, these coatings are referred to as Cr, Cr_0.91_Ni_0.09_, Cr_0.83_Ni_0.17_, and Cr_0.73_Ni_0.27_, respectively. All the Cr-Ni alloy coatings possessed a thickness of approximately 5.4 μm, while the pure Cr coating exhibited a larger thickness.

### 2.2. Au-Ions Irradiation

**Figure 1:**
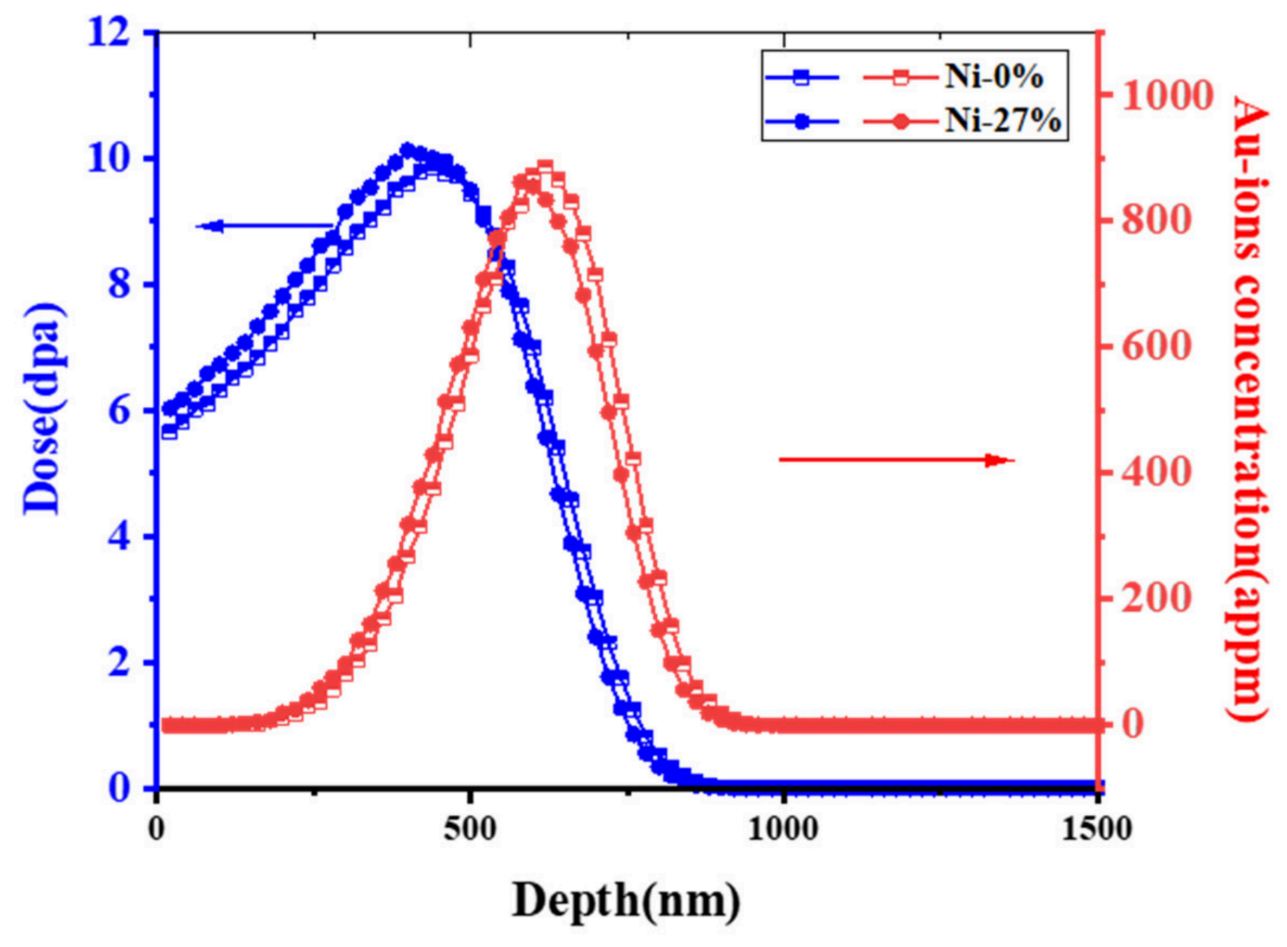
SRIM-2008 simulated damage and concentration of Cr and Cr_0.73_Ni_0.27_ coatings by Au^2+^ to a fluence of 2.3 × 10^15^ ion/cm^2^.

The irradiation experiments were conducted on a tandem electrostatic accelerator platform at the Institute of Nuclear Science and Technology, Sichuan University [28]. The irradiations were performed at room temperature using 6 MeV Au ions with a total fluence of 2.3 × 10^15^ ion/cm^2^, aimed at investigating the effects of Au ion irradiation on the microstructure and mechanical properties of four different CrNi coatings. Radiation damage dose and implanted ion concentration as a function of irradiation depth were simulated using the “Quick Kinchin-Pease” mode in the SRIM (2013) software [29], with displacement threshold energies set to 40 eV for both Cr and Ni [30]. Figure 1 presents the SRIM simulation results for the Cr and Cr_0.73_Ni_0.27_ coatings irradiated with 6 MeV Au ions at a fluence of 2.3 × 10^15^ ion/cm^2^. The maximum injection depth of Au ions was found to be close to 900 nm, and the maximum concentration was observed at a depth of 650 nm. At the fluence of 2.3 × 10^15^ ion/cm^2^, the peak radiation doses for the Cr and Cr_0.73_Ni_0.27_ coatings were 9.8 dpa and 10.1 dpa, respectively. Peak damage occurs at a depth of 450 nm within the coating; this is the damage zone where preliminary voids or dislocations form [31,32].

### 2.3. Microstructure and Mechanical Characterization

The crystal structures of the CrNi coatings, both before and after Au ion irradiation, were systematically investigated by X-ray diffraction (XRD, DX-2700, Dandong Fangyuan, Dandong, China) employing Cu Kα radiation (λ = 1.5406 Å). The XRD scans were performed in a continuous mode over the 2θ range of 20–90° with a step size of 0.02° and a scanning speed of 0.02°·s^−1^. Microstructural features, including surface morphology and cross-sectional characteristics of the coatings, were examined using a field-emission scanning electron microscope (FE-SEM, Thermo Scientific Apreo 2C) operated at an accelerating voltage of 10 kV. For cross-sectional analysis, the specimens were carefully mounted in epoxy resin at room temperature to preserve interface integrity. The mounted samples were then mechanically ground with 3000-grit SiC paper and meticulously polished using diamond suspension to achieve a mirror-like finish, ensuring a deformation-free surface for accurate observation. Site-specific TEM lamellae were prepared via focused ion beam (FIB) milling in an FEI Scios DualBeam system. To protect the region of interest from ion damage, a Pt protective layer was deposited in situ prior to milling. The resulting thin foils were subsequently analyzed using high-resolution transmission electron microscopy (HRTEM, Talos F200X, FEI, Thermo Scientific) operated at 200 kV. Complementary microstructural and chemical analyses were conducted using selected-area electron diffraction (SAED) and energy-dispersive X-ray spectroscopy (EDS) with a super-X EDS detector, enabling comprehensive investigation of irradiation-induced microstructural evolution and elemental distribution in the CrNi coatings with varying Ni contents. Nanoindentation tests were performed using an Anton Paar NHT2 system equipped with a Berkovich diamond indenter to evaluate the mechanical properties of the coatings prior to and post-irradiation. The maximum indentation depth was strictly maintained between 1/7 and 1/10 of the total film thickness to preclude substrate effect. A minimum of five valid indentations were performed on each sample to ensure statistical reproducibility.

## 3. Results and Discussion

### 3.1. Initial Coating Microstructure Characterization

**Figure 2:**
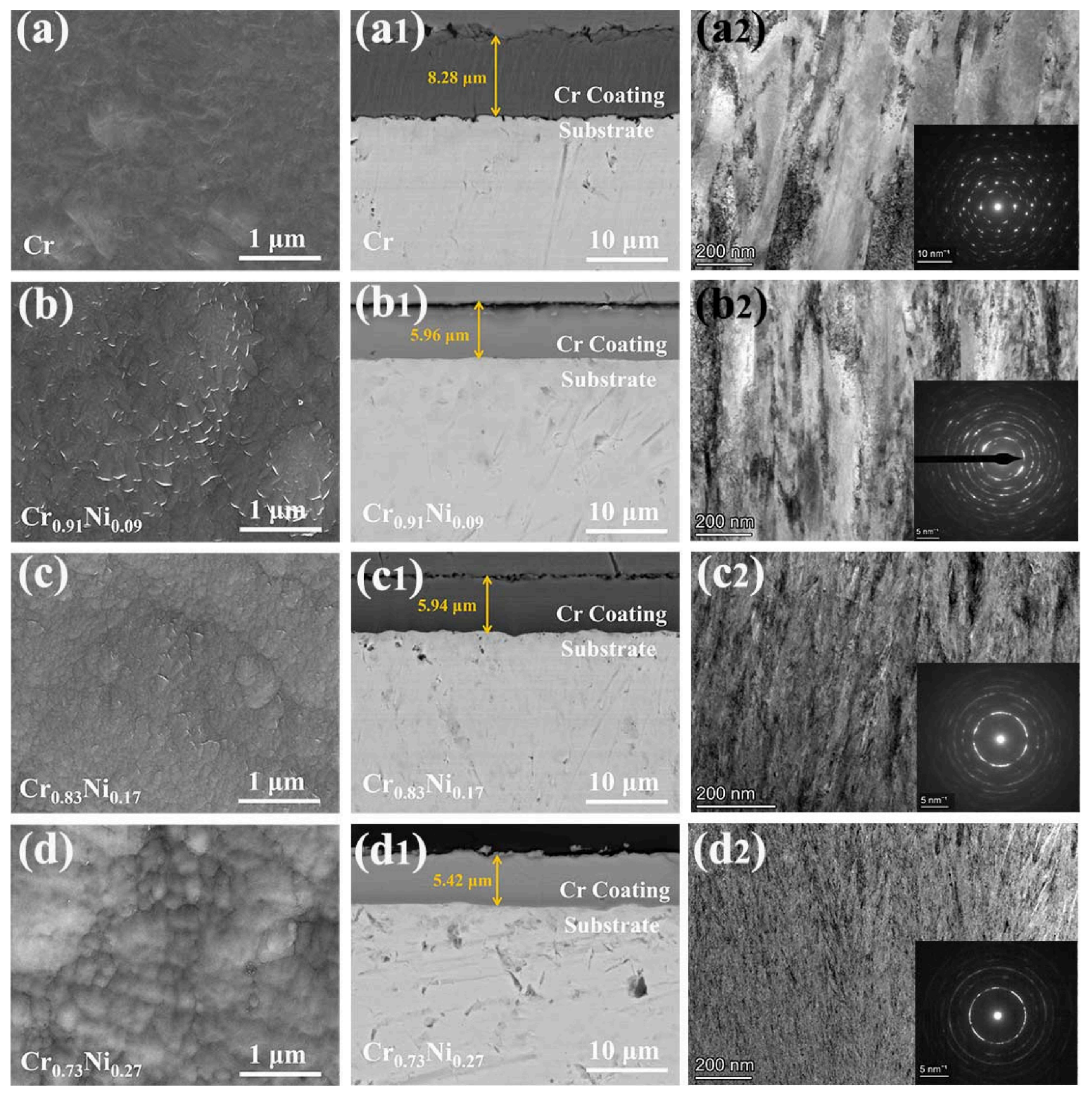
Surface and cross-sectional morphologies of the as-deposited coatings: (**a**,**a1**) Cr, (**b**,**b1**) Cr, Cr_0.91_Ni_0.09_, (**c**,**c1**) Cr_0.83_Ni_0.17_, (**d**,**d1**) Cr_0.73_Ni_0.27_. Bright-field TEM images and selected area electron diffraction (SAED) patterns of the Cr coatings with varying Ni contents: (**a2**) Cr, (**b2**) Cr, Cr_0.91_Ni_0.09_, (**c2**) Cr_0.83_Ni_0.17_, (**d2**) Cr_0.73_Ni_0.27_.

Figure 2 displays the surface morphologies and microstructures of the CrNi coatings with different Ni contents. As observed from the surface morphologies of the as-deposited CrNi coatings in Figure 2a–d, all the coatings present uniform and dense microstructures, demonstrating favorable deposition quality. Notably, the surface morphology of the coatings exhibits an obvious dependence on the Ni content, and the surface roughness increases with rising Ni concentration. This phenomenon can be primarily attributed to the intrinsic crystal structures of Cr (body-centered cubic, BCC) and Ni (face-centered cubic, FCC). The incorporation of Ni atoms into the BCC lattice of Cr induces remarkable lattice distortion and solid-solution strengthening, accompanied by an increase in the internal compressive stress within the coatings [33,34]. The degree of lattice distortion intensifies with the increase in Ni atomic fraction in the Cr matrix, leading to an elevation of the internal energy of the coatings. To release the accumulated energy, the coatings tend to relieve stress via grain boundary formation, dislocation tangling and surface undulation, which is macroscopically manifested as increased surface roughness [35]. Cross-sectional morphologies of the CrNi coatings with varying Ni contents are illustrated in Figure 2(a1–d1). The thicknesses of the Cr, Cr_0.91_Ni_0.09_, Cr_0.83_Ni_0.17_, and Cr_0.73_Ni_0.27_ coatings are 8.28 μm, 5.96 μm, 5.94 μm and 5.42 μm, respectively. The reduced coating thickness after Ni alloying is mainly attributed to the difference in sputtering yields between Ni and Cr during the magnetron sputtering process, which lowers the overall deposition rate [36]. TEM characterization was further employed to reveal the differences in the microstructure of the four coatings. Figure 2(a2–d2) present the bright-field TEM images and corresponding selected area electron diffraction (SAED) patterns of the Cr coatings with varying Ni contents. Overall, all the coatings exhibit a dense structure without pores or cracks. Notably, the pure Cr coatings consist of columnar grains growing perpendicularly to the zirconium alloy substrate [37], and the grain size decreases gradually with increasing Ni content. The grain refinement induced by Ni addition originates from three dominant mechanisms: elevated nucleation density, suppressed atomic diffusion and grain boundary pinning, through which Ni solute atoms greatly modify the crystallization process of Cr coatings [34]. It is worth mentioning that when the Ni content rises to 17%, the grain boundaries become indistinct, indicating deteriorated crystallinity, which is also verified by the corresponding diffraction results.

**Figure 3:**
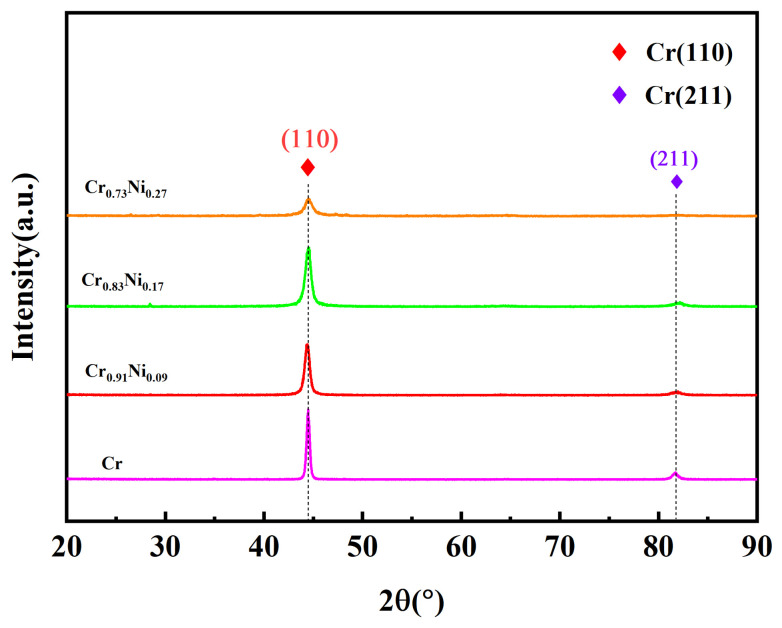
XRD patterns of pre-irradiated samples.

XRD analysis was performed to investigate the phase structures of the CrNi coatings with various Ni contents before irradiation, as presented in Figure 3. The positions and intensities of the two diffraction peaks are well indexed to the (110) and (211) crystal planes of the chromium phase. Diffraction peak broadening is observed with increasing Ni content, indicating a marked refinement of the grain size. This is consistent with the findings of Naka [22], who confirmed that Ni addition can effectively refine Cr grain size. As the Ni content reaches 27%, the intensity of the (110) peak decreases and the (211) peak disappears completely, demonstrating a deterioration in the crystallinity of the coating [38,39].

### 3.2. Evolution of Coating Microstructure Under Irradiation

**Figure 4:**
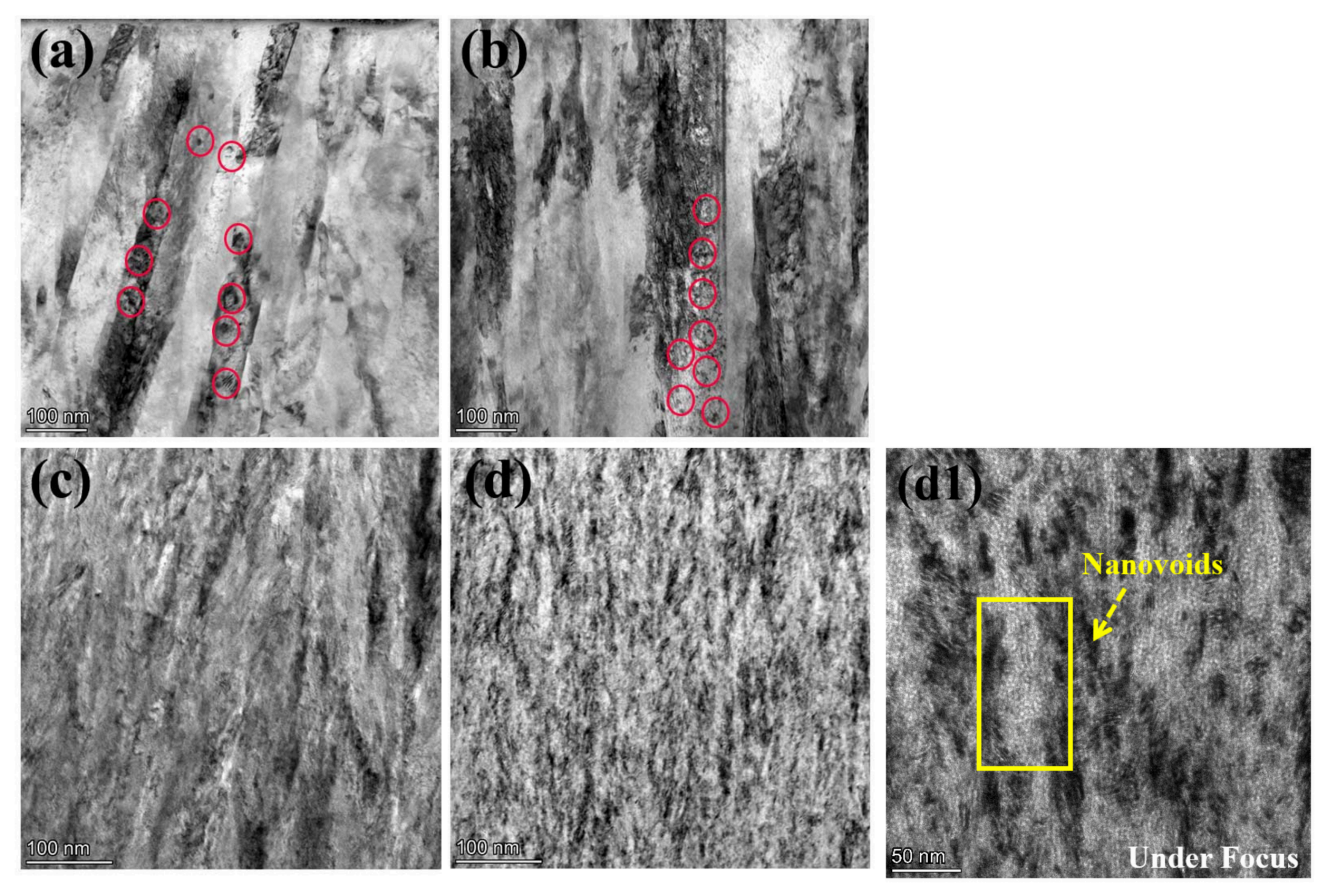
Bright-field TEM images of irradiated: (**a**) Cr, (**b**) Cr_0.91_Ni_0.09_, (**c**) Cr_0.83_Ni_0.17_, (**d**) Cr_0.73_Ni_0.27_. BF underfocus magnified micrograph post irradiation: (**d1**) Cr_0.73_Ni_0.27_.

**Figure 5:**
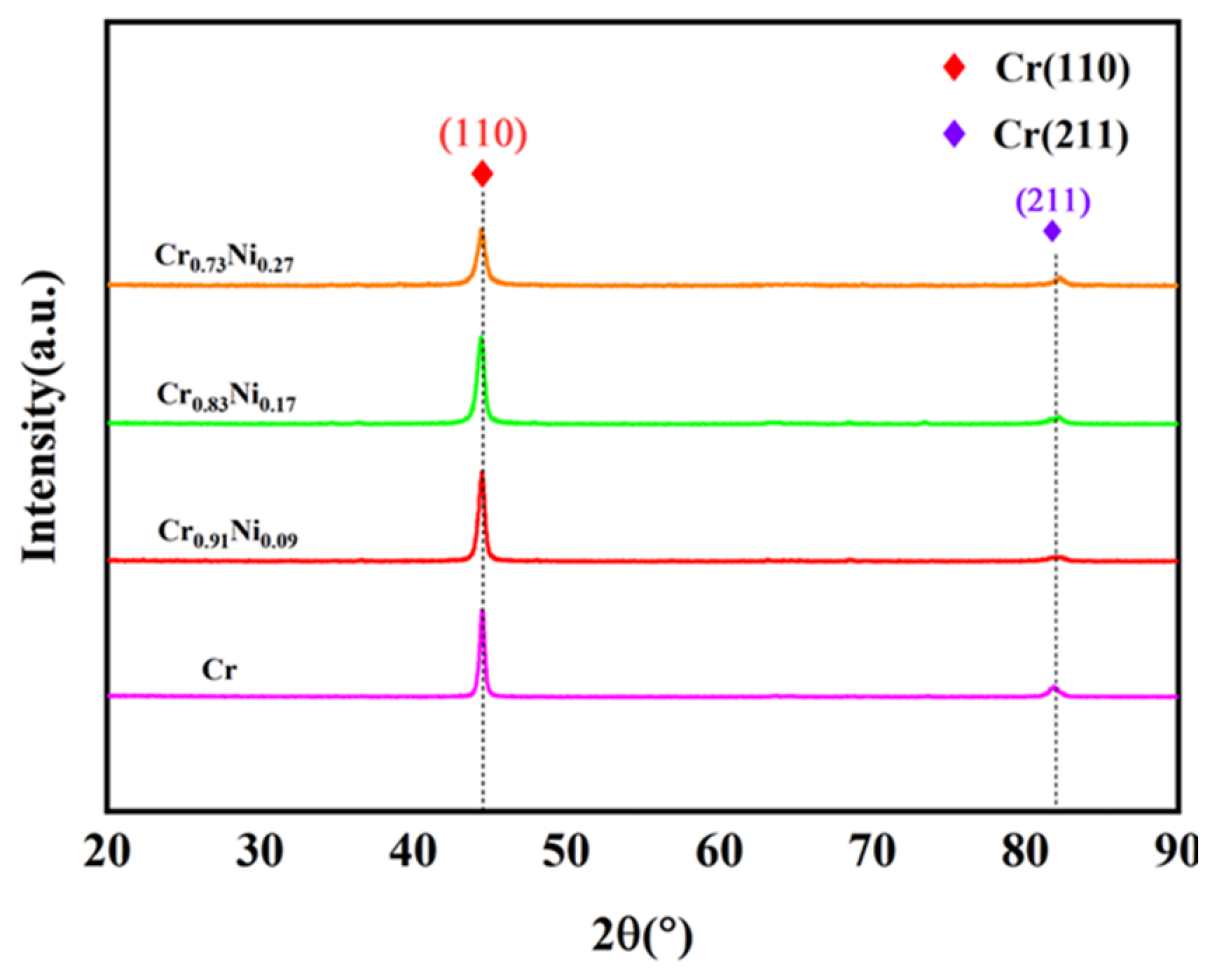
XRD patterns of post-irradiated samples.

Figure 4 presents TEM images of the peak damage region in the CrNi coatings with different Ni contents after Au-ion irradiation. In the pure Cr and Cr_0.91_Ni_0.09_ coatings, numerous black-spot defects (red circles) are observed, which are small interstitial- or vacancy-type clusters generated by cascade collisions and serve as precursors for dislocation loop nucleation. As Ni content increases to Cr_0.73_Ni_0.27_, dislocation loops decrease significantly, accompanied by the formation of dislocation-free zones and irradiation-induced nanovoids (yellow square) [40,41]. The underlying mechanisms are as follows. High Ni reduces the stacking fault energy and induces local lattice distortion, which, on one hand, suppresses the aggregation of interstitial clusters and their transformation into mobile dislocation loops, and on the other hand, enhances interstitial-vacancy recombination efficiency. The resulting excess vacancies aggregate via diffusion to form nanovoids. Meanwhile, local defect annihilation during dynamic recovery gives rise to dislocation-free zones [42,43]. The XRD results (Figure 5) further reveal that irradiation reduces the peak intensities of pure Cr, indicating degraded crystallinity. In contrast, the Cr_0.73_Ni_0.27_ coating exhibits increased peak intensities after irradiation, which is attributed to “irradiation-induced crystallization”; the initially highly disordered structure undergoes defect annihilation, atomic rearrangement, and enhanced short-range order (SRO) under the localized thermal spike effect of cascade collisions.

Figure 5 displays the XRD patterns of the four films after irradiation. Compared to the pristine counterparts (Figure 2), the (110) diffraction peak of the Cr_0.73_Ni_0.27_ coatings becomes significantly more intense, and the (211) plane reappears. The as-deposited coating is Cr-rich and exhibits poor crystallinity, with severe lattice distortion and a high density of defects. As a result, its XRD peaks are broadened and weak, and the (211) peak may even be indiscernible from the background. Upon high-energy Au ion irradiation, the thermal spike effect and collision cascade induced by ion energy deposition promote atomic rearrangement in localized regions [44,45], driving a transition from short-range to long-range order, i.e., irradiation-induced crystallization [43,46]. Concurrently, point defects undergo recombination and annihilation, and internal stresses are released, leading to enhanced crystalline quality. These changes are manifested by the increased intensity of the (110) peak and the reappearance of the (211) peak. It is worth noting that the probing depth of conventional X-ray diffraction typically ranges from submicron to several micrometers, depending on the material and the incident angle; therefore, the XRD data presented here mainly reflect the structural evolution in the near-surface region of the films.

**Figure 6:**
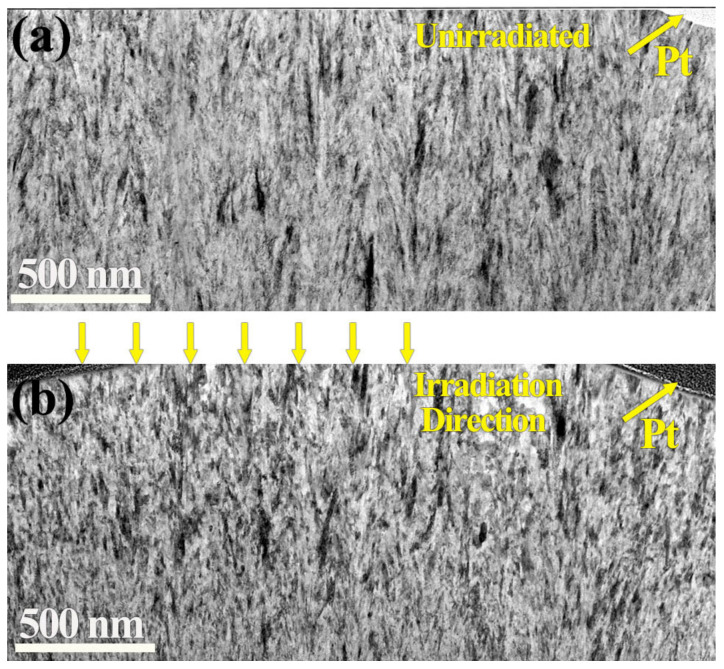
Surface TEM images of Cr_0.73_Ni_0.27_ coating: (**a**) pre-irradiation and (**b**) post-irradiation.

To further analyze the microstructural evolution of the high-Ni CrNi coatings after irradiation, Figure 6 presents the near-surface TEM images of the Cr_0.73_Ni_0.27_ coating before and after irradiation. Evidently, the crystallinity of the coating surface is remarkably improved after irradiation, which is consistent with the XRD results. This demonstrates that the highly disordered structure of the high-Ni coating undergoes recrystallization during ion irradiation [47,48].

### 3.3. Effect of Irradiation on Mechanical Properties

**Figure 7:**
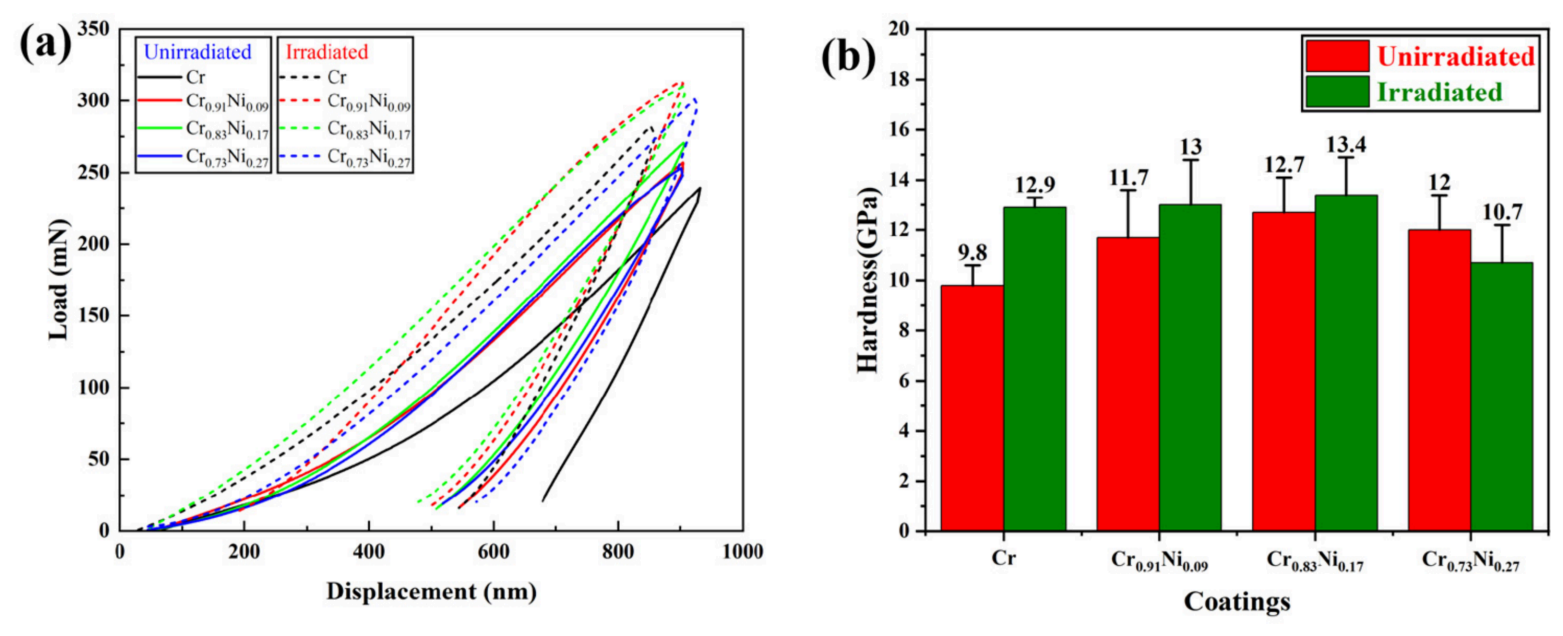
(**a**) The typical nanoindentation Load–Displacement curves and (**b**) hardness as a function of irradiation dose. Cr, Cr_0.91_Ni_0.09_, Cr_0.83_Ni_0.17_, and Cr_0.73_Ni_0.27._

The irradiation-induced hardening of the CrNi coatings with varying Ni content was evaluated by nanoindentation performed before and after irradiation, with the indentation direction parallel to the ion beam. Figure 7a presents typical load–displacement curves for the CrNi coatings with different Ni contents, reflecting variations in hardness. Figure 7b summarizes the nano-hardness evolution of all the coatings prior to and subsequent to irradiation, as determined by the Oliver & Pharr method. Prior to irradiation, the pure Cr coating exhibits a hardness of 9.8 GPa. The coating with 17% Ni exhibits the highest hardness of 12.7 GPa, while further increasing Ni to 27% reduces the hardness to 12.0 GPa. This trend arises from the competition between grain refinement strengthening [49] and crystallinity deterioration. At low Ni concentrations, the incorporation of Ni refines the columnar grain structure, and the combined effects of grain refinement and solid-solution strengthening progressively improve the coating hardness [50]. Nevertheless, excessive Ni content (27%) leads to a remarkable degradation in crystallinity, reduced lattice order, and abundant internal defects, which weakens the grain refinement effect. Concurrently, the presence of a relatively soft Ni phase introduces a hardness dilution effect, resulting in an overall reduction in coating hardness.

After ion irradiation, the pure Cr coating exhibits the most pronounced irradiation hardening, with a hardness increase of 3.1 GPa relative to its pre-irradiated state. For the CrNi coatings, the post-irradiation hardness first increases and then decreases with rising Ni content, reaching a maximum of 13.4 GPa at 17% Ni, and subsequently decreasing to 10.7 GPa at 27% Ni. Notably, excessive Ni alloying leads to irradiation-induced softening in the Cr coating. The underlying mechanisms are summarized as follows. At low Ni contents (0%, 9%, and 17%), the coatings are predominantly composed of columnar grains. The high density of irradiation-induced defects, such as dislocation loops and defect clusters, results in significant irradiation hardening [51]. At 17% Ni, the interaction between solute atoms and irradiation-induced defects leads to the formation of solute–defect complexes, providing additional synergistic strengthening and yielding the peak hardness [52]. As the Ni content increases to 27%, the initial crystallographic integrity of the coating deteriorates. Under irradiation, local disordering or grain boundary weakening may occur, thereby diminishing the defect-induced strengthening effect and reducing the hardness increment [53]. It is well established that irradiation hardening is a common phenomenon in metallic materials, arising from the formation of dislocations, precipitates, and voids. However, when the Ni content reaches 27%, the coating exhibits poor initial crystallinity and a nearly disordered structure. Irradiation further disrupts the remaining ordered regions and introduces free volume and nanoscale voids, leading to irradiation softening [53]. Additionally, the high content of the relatively soft Ni phase dilutes the intrinsic strength of the coating. Consequently, the post-irradiation hardness drops sharply to 10.7 GPa, which is even lower than that of the irradiated pure Cr coating.

These results demonstrate that moderate Ni alloying (~17%) effectively improves the irradiation hardening resistance of the CrNi coatings. Nevertheless, excessive Ni content severely degrades the mechanical properties due to irradiation-induced disordering and soft-phase dilution. Such irradiation softening is attributed to the recovery of irradiation-modified deformation substructures and the trapping of interstitial solutes by irradiation defects. Chen et al. [54] characterized arc-ion-plated Cr coatings after 5 MeV proton irradiation and reported analogous irradiation softening phenomena. Their studies revealed that irradiation-induced defects in Cr coatings mainly consist of voids, dislocations and vacancy–bubble complexes. Notably, irradiation softening was also detected in arc-ion-plated chromium coatings, which is considered to originate from deformation recovery and defect recombination of inherent structural imperfections and irradiation-induced defects.

## 4. Conclusions

In this study, 6 MeV Au ions were adopted to irradiate CrNi coatings with various Ni contents for investigating the effects of Ni concentration on the irradiation response of Cr-based coatings. The evolutions of surface morphology, microstructure and irradiation hardening behavior of the coatings were systematically analyzed. The conclusions corresponding to the three predefined research objectives are summarized as follows:

1. With increasing Ni content, the surface roughness of CrNi coatings increases, the columnar grain size is refined, and the coating thickness decreases slightly from 8.28 μm to 5.42 μm. Coatings with low Ni content maintain favorable crystallinity. When the Ni content rises to 27%, indistinct grain boundaries, diffraction peak broadening and reduced peak intensity are observed, demonstrating remarkable deterioration in crystallinity and a tendency toward structural disorder.
2. Distinct from the typical dislocation loop accumulation and weakened diffraction peaks in low-Ni coatings after irradiation, few dislocation loops and dislocation-free regions are detected in the 27% Ni coating, accompanied by abnormally enhanced XRD diffraction peak intensity. The TEM results further verify the higher surface crystallinity after irradiation. This phenomenon originates from the highly disordered initial structure of the coating. Driven by the thermal spike effect and collision cascades of high-energy Au ions, local recrystallization, intrinsic defect annihilation and internal stress relief occur, which reorganize atomic arrangements toward long-range order, namely irradiation-induced crystallization.
3. Before irradiation, the coating hardness rises first and then drops with increasing Ni content, reaching a maximum of 12.7 GPa at 17% Ni, which is mainly attributed to the combined effects of grain refinement strengthening and solid-solution strengthening. The hardness decline of the 27% Ni coating arises from crystallinity degradation. After irradiation, the hardness reaches its peak of 13.4 GPa at 17% Ni, and continuously decreases to 10.7 GPa for the 27% Ni coating, accompanied by irradiation softening. Moderate Ni alloying (17%) achieves optimal irradiation hardening via the interaction between solute atoms and irradiation defects.

## Data Availability

The original contributions presented in this study are included in the article. Further inquiries can be directed to the corresponding authors.
